# Submarine Telegraphs

**Published:** 1865-08

**Authors:** 


### SUBMARINE TELEGRAPHS.

For 22 or more years, experiments on this subject have been made upon both sides of the Atlantic. In 1843, Samuel Colt, of revolver fame, laid and operated a cable running from the city of New York to Long Island in the bay. In 1856, connected coils of wire, two thousand miles long, covered with gutta percha, transmitted signals to the number of two hundred and seventy per minute, but, upon being submerged, the signals decreased in intensity and grave defects were discovered which required time and study for correction.

The following table, prepared by scientific observers, and compared with other tables extant, gives, perhaps, as reliable figures as have been prepared:

| Date when laid...... | From | To | Time the Cables have been working...... | Depth of Water in Fathoms.......... | Length of Cable in Statute Miles....... | Length of Insulated Wire in Statute Miles |
|---|---|---|---|---|---|---|
| | | | Yrs. | | | |
| 1851 | Dover.................. | Calais............ | 12 | .... | 27 | 108 |
| 1853 | Denmark ............. | Across Belt........ | 10 | .... | 18 | 54 |
| 1853 | Dover.... ............. | Ostend............ | 10 | .... | 80½ | 483 |
| 1853 | Frith of Forth........... | ................ | 10 | .... | 6 | 24 |
| 1853 | Portpatrick.............. | Donaghadee........ | 10 | .... | 25 | 150 |
| 1853 | Across the River Tay.... | ................... | 10 | .... | 2 | 8 |
| 1854 | Portpatrick.............. | Whitehead......... | 9 | .... | 27 | 162 |
| 1854 | Sweden...... ......... | Denmark.......... | 9 | 14 | 12 | 36 |
| 1854 | Italy .... ............. | Corsica........... | 9 | 325 | 110 | 660 |
| 1854 | Corsica ................ | Sardinia..... ..... | 9 | 30 | 10 | 60 |
| 1855 | Egypt ................. | .............. ... | 8 | .... | 10 | 40 |
| 1855 | Italy .. .... ......... | Sicily............. | 8 | 20 | 5 | 15 |
| 1856 | Newfoundland........... | Cape Breton........ | 7 | 360 | 85 | 85 |
| 1856 | Prince Edward .......... | New Brunswick..... | 7 | 14 | 12 | 12 |
| 1856 | Strait Canso............ | Cape Breton, N. S... | 7 | .... | 1½ | 41½ |
| 1857 | Norway............. ... | Across Flords....... | 6 | 300 | 40 | 49 |
| 1857 | Across the mouths of the.. | Danube .... ... | 6 | .... | 3 | 3 |
| 1857 | Ceylon.................. | Mainland India ..... | 6 | ... | 30 | 30 |
| 1858 | Italy.................... | Sicily............ | 5 | 60 | 8 | 8 |
| 1858 | England................ | Holland.......... | 5 | 30 | 140 | 560 |
| 1858 | England................ | Hanover... ....... | 5 | 30 | 280 | 500 |
| 1858 | Norway ........... .... | Across Flords ..... | 5 | 300 | 16 | 16 |
| 1858 | South Australia .......... | King's Island....... | 5 | 45 | 140 | 140 |
| 1858 | Ceylon................... | India........... . | 5 | 45 | 30 | 30 |
| 1859 | Alexandria............... | ................... | 4 | .... | 2 | 8 |
| 1859 | England................ | Denmark. ........ | 4 | 30 | 368 | 1104 |
| 1859 | Sweden.................. | Gotland........... | 4 | 80 | 64 | 64 |
| 1859 | Folkestone.............. | Boulogne.......... | 4 | 32 | 24 | 144 |
| 1859 | Across Rivers........... | In India..... ..... | 4 | .... | 10 | 10 |
| 1859 | Malta.................. | Sicily.... ....... | 4 | 79 | 63 | 60 |
| 1859 | England ................ | Isle of Man........ | 4 | 30 | 36 | 36 |
| 1859 | Suez................... | Jubal Island........ | 4 | .... | 220 | 220 |
| 1859 | Jersey.................. | Pirou, France...... | 3 | 15 | 21 | 21 |
| 1859 | Tasmania................ | Bass Strait......... | 3½ | .. | 240 | 240 |
| 1860 | France.................. | Algiers........... | 2½ | 1585 | 520 | 520 |
| 1860 | Corfu .................. | Otranto.......... | 2½ | 1000 | 90 | 90 |
| 1860 | Denmark ................ | (Great Belt)....... | 3 | 18 | 28 | 126 |
| 1860 | Dacca.................. | Pegu ............ | 3 | ... | 116 | 116 |
| 1860 | Barcelona.... .......... | Mahon............ | 3 | 1400 | 180 | 180 |
| 1860 | Minorca......... ..... | Majora........... | 3 | 200 | 35 | 70 |
| 1860 | Iviza.............. .... | Majorca.......... | 3 | 500 | 74 | 148 |

| Date when Laid.... | From | To | Time the Cables have been working ...... | Depth of Water in Fathoms.......... | Length of Cable in Statute Miles...... | Length of Insulated Wire in Statute Miles |
|---|---|---|---|---|---|---|
| 1860 | St. Antonio ............. | Iviza........ ..... | 3 | 450 | 76 | 152 |
| 1861 | Norway ................. | Across Foirds...... | 2¼ | 300 | 16 | 16 |
| 1861 | Toulon... .............. | Corsica........... | 2¼ | 1550 | 185 | 195 |
| 1861 | Holyhead................ | Howth, Ireland .... | 2 | .... | 64 | 64 |
| 1861 | Malta .................. | Alexandria ........ | 2½ | 420 | 1535 | 1535 |
| 1861 | Newhaven................ | Dieppe .. ........ | 2 | .. | 80 | 320 |
| 1862 | Pembroke, W ............ | We ford, Ireland... | 1½ | 28 | 63 | 252 |
| 1862 | Fiith of Forth.......... | .................. | 1 | .. | 6 | 24 |
| 1862 | England................. | Holland........... | 0¼ | 30 | 130 | 520 |
| 1862 | Across the River Tay...... | .................. | 1 | .. | 2 | 8 |
| 1863 | Sardinia ................. | Sicily ............ | 0½ | 1200 | 243 | 243 |
| | Total.... ...................... ............ | | | | 5625 | 9783¼ |

Of the cables named in the above table, but few can be called successful, and the application of electrical science to submarine routes of communications is comparatively in its infancy.

The three great attempts at success which have proved their claim to energy, perseverance and glory, even in spite of their want of success, are the Atlantic cables of 1857, 1858 and 1865, each conducted by our countryman, Cyrus W. Field.

The trial of 1857 was made by the Niagara and Agamemnon, which vessels each took half of the cable. The former was to lay the Irish half and the latter the American. On the 5th of August the shore end was laid at Valentia. On the 11th, the Niagara, after laying 334 miles, parted the cable in a swell, leaving but 759 miles on board of her. There were on board the Agamemnon 1,847 miles, or a bare surplus on the two vessels of 207 miles over 1,634 miles, the entire distance between the two countries, hence the attempt was abandoned.

In 1858, the same vessels met in mid-ocean, spliced their two ends of cable, and sailed apart forty miles, when, on the 26th of June, the cable parted. Two days after they again met, united their cables, and after laying 145 miles, the cable again parted. The vessels now recoaled at Queenstown, and again went to mid-ocean and began anew their work, this time with entire success. The *cable was laid*, and the possibility of communication between the continents was demonstrated. We all remember how the dispatch of Cyrus W. Field on the 5th day of August, 1858, electrified the country, and how the people were fairly beside themselves with joy and admiration.

For nearly two months the two worlds talked to each other with mouth to ear. One hundred and twenty-nine messages were sent from Valentia to New England, and two hundred and seventy-one in the other direction. Thus were four thousand three hundred and fifty-nine words exchanged. The message of Queen Victoria to the President contained ninety-nine words, and occupied in its reception at Newfoundland sixty-seven minutes. High hopes were formed but these were blighted. The fate of the cable of 1858 is thus told by an exchange:

"On September 24th, DeSauty verified the worst apprehensions that had been formed of the failure of the cable, by informing the country that 'nothing intelligible had been received from Valentia since September 1st.' This could hardly have resulted from any injury to the cable, for afterwards, on October 2d, in the course of their experiments, a last communication was received and imperfectly read as follows: 'Two hundred and forty t——k——Daniells now in circuit.' It afterwards appeared that the full message was 'Two hundred and forty trays and seventy-two liquid Daniells now in circuit.' The power thus employed was one thousand times what would be required in an ordinarily well insulated conductor to give perfect signals. Further efforts were therefore abandonod as hopeless. The cost of the cable thus laid was $1,256,250—the total expenditures of the company were $1,834,500."

The cable of 1865 was 2600 miles long and weighed 5,000 tons, and is one and one-eighth inches in diameter.

"The 'core,' or conductor is made of seven fine copper wires twisted into a single strand, add insulated by a compound known as Chatterton's. Around this 'core' are four layers of gutta percha, insulated with the same compound, and in turn enclosed by eleven strong iron wires, each one of which is carefully wound with Manilla thread, and saturated with tar, thus at once protecting the gutta percha and adding strength to the cable. Its strength is equal to a bearing strain of eleven and three-quarters tons. As compared with the tensile strength of the cable of 1858, it is several times greater, while the electrical tests show the present wire to be infinitely superior to that of the cable of 1858.—*N. W. Christian Advocate.*

